# *Pseudomonas* species isolated via high-throughput screening significantly protect cotton plants against verticillium wilt

**DOI:** 10.1186/s13568-020-01132-1

**Published:** 2020-10-28

**Authors:** Xiaoyuan Tao, Hailin Zhang, Mengtao Gao, Menglin Li, Ting Zhao, Xueying Guan

**Affiliations:** 1 College of Agriculture and Biotechnology, Zhejiang University, Hangzhou, 310058 China; 2 State Key Laboratory of Crop Genetics and Germplasm Enhancement, Cotton Hybrid R & D Engineering Center (the Ministry of Education), College of Agriculture, Nanjing Agricultural University, Nanjing, 210095 China

**Keywords:** Rhizosphere, *Pseudomonas*, Verticillium wilt, Biocontrol, 7-hydroxytropolone

## Abstract

*Verticillium* wilt (VW) caused by *Verticillium dahliae* is a devastating soil-borne disease that causes severe yield losses in cotton and other major crops worldwide. Here we conducted a high-throughput screening of isolates recovered from 886 plant rhizosphere samples taken from the three main cotton-producing areas of China. Fifteen isolates distributed in different genera of bacteria that showed inhibitory activity against *V. dahliae* were screened out. Of these, two *Pseudomonas* strains, *P. protegens* XY2F4 and *P. donghuensis* 22G5, showed significant inhibitory action against *V. dahliae*. Additional comparative genomic analyses and phenotypical assays confirmed that *P. protegens* XY2F4 and *P. donghuensis* 22G5 were the strains most efficient at protecting cotton plants against VW due to specific biological control products they produced. Importantly, we identified a significant efficacy of the natural tropolone compound 7-hydroxytropolone (7-HT) against VW. By phenotypical assay using the wild-type 22G5 and its mutant strain in 7-HT production, we revealed that the 7-HT produced by *P. donghuensis* is the major substance protecting cotton against VW. This study reveals that *Pseudomonas* specifically has gene clusters that allow the production of effective antipathogenic metabolites that can now be used as new agents in the biocontrol of VW.

## Key points

- *Pseudomonas* spp. isolated from high-throughput screening showed the most influential activities to multiple strains of *V. dahlia*.
- *P. protegens* XY2F4 and *P. donghuensis* 22G5 showed that *Pseudomonas* spp. have developed specific mechanisms against *V. dahlia*.
- 7-hydroxytropolone produced by *P. donghuensis* is the major ingredient to protect cotton against verticillium wilt.

## Introduction

Cotton verticillium wilt (VW) is a singularly destructive fungal disease caused by *Verticillium dahliae*, which is regarded as “the cancer of cotton”. The *V. dahliae* fungus invades the vascular system through the roots and soon causes systemic infection, leading to a series of symptoms including leaf chlorosis, necrosis or wilting, leaf or boll abscission, and even plant death. VW-related damage results in reduced cotton yield and lower fiber quality in agricultural production (Wang et al. 2016). Currently, around 50% of the cotton planting area in China (2.5 million hectares) is VW-infected, leading to direct economic losses of about 250–310 million USD annually (Wang et al. 2016). Disease management mainly includes crop rotation to non-host plants, fungicide fumigation and breeding of resistant cultivars (Klosterman et al. 2009). Crop rotation is a preventative, but not curative disease management strategy since *V. dahliae* can survive for extremely long periods of time in the soil as microsclerotia even in the absence of a suitable host (Termorshuizen 1995). Disease control of VW using fungicide fumigation is effective, but expensive and environmentally unfriendly (Jordan 1972). Breeding of cultivars with broad-spectrum resistance is considered to be one of the most practicable and effective approaches. However, it is difficult to apply biotechnology to breed VW-resistant cotton due to the lack of resistance markers in cotton germplasm, only a few commercial upland cotton cultivars have developed with moderate levels of VW resistance (Zhang et al. 2012a).

Rhizobacteria have great potential to improve sustainable agricultural practices due to their influence on growth, yield, nutrient uptake, and biotic/abiotic tolerance of crops. Beneficial rhizobacteria are able to colonize the rhizosphere (the root surface or intercellular spaces of plants), which impacts the plant by delivering biocontrol and other beneficial factors (Lugtenberg et al. 2001). To date, multiple isolates from genera of *Enterobacter* (Li et al. 2012a), *Bacillus* (Li et al. 2012b; Zhang et al. 2018), *Serratia plymuthica* (Vleesschauwer 2007), *Streptomyces* (Xue et al. 2013) and *Pseudomonas* (Erdogan and Benlioglu 2010) have documented biocontrol activities against *V. dahliae* in *in planta* assays. Thus, beneficial rhizobacteria with inhibitory action against *V. dahliae* are promising biocontrol agents for the management of VW in cultivated cotton (Tjamos et al. 2000). However, the specific mechanisms underlying the biocontrol of VW have yet to be determined. In this research we employed a high-throughput screening for inhibitory isolates and comparative genomic analysis to uncover the mode of action of two new *Pseudomonas* strains with significant *V. dahliae* inhibitory capacity. This study charts a path toward the development of probiotics and active ingredients for biocontrol agents (BCAs) to ameliorate cotton VW disease.

## Materials and methods

### Plant culture

Upland cotton (*Gossypium hirsutum*) cultivars Texas Marker-1 (TM-1) and Junmian1 were grown in soil consisting of 25% vermiculite and 75% artificial soil at 28 °C with a 16 h/8 h light/dark cycle in growth chambers. One-week old seedlings were used in the planta assays.

### *V. dahliae* culture

Highly virulent strains of *V. dahliae*, including V07DF2, V08DF2, V15QY1, and V991 were gifts from Institute of Plant Protection, Jiangsu Academy of Agricultural Sciences. The highly toxic and defoliant wild type pathogenic *V. dahliae* strain V991 was used *in planta* assay (Sun et al. 2013; Zhang et al. 2012b). *V. dahlia* strains were cultured on Czapek agar plates at 28 °C for 4–5 days after which 5 mL liquid Czapek was dispensed into Petri plates to collect the conidia. The conidia suspension was then transferred to 100 mL liquid Czapek and cultured for 7 days until cell density reached OD_600_ = 2, contains ~ 3 × 10^6^ conidia/mL. Finally, the conidia were filtered through a 500-micron sieve for use in inoculation assays. The *V. dahliae* strain stock was composed of the conidia suspensions with 20% glycerol.

### Bacteria isolation and culture

Bacterial isolates were recovered from 886 plant rhizosphere samples taken from the three main cotton-producing areas of China (the Yangtze river basin, the Yellow River basin and Xinjiang) (Additional file 1: Table S1). The samples were placed in separate, labeled 50 mL tubes filled with enough ddH_2_O to ensure that they were completely submerged, and then tubes were shaken approximately 4–5 times to mix. 800 µL of the mixture was aliquoted for gradient dilution (10^−1^, 10^−2^, 10^−3^, 10^−4^ and 10^−5^). 1:10^3^ or 1:10^4^ was considered a suitable dilution ratio and 100 µL solution was plated on LB media and inoculated overnight at 30 °C. Plates were stored at 4 °C for 3 days in order to enhance the formation of fluorescent pigments in bacterial colonies.

### High-throughput screening for bacterial isolates with inhibitory action against *V. dahliae*

A plate assay was performed to screen isolates for inhibitory action against *V. dahliae*. In a 10-mL tube, 6 mL top agar (0.8% w/v) was cooled to less than 50 °C and gently mixed with 60 µL V*. dahliae* V991 culture stock (OD_600_ = 2), resulting in an initial density of 0.02/mL at OD_600_ in the top agar. This solution was then quickly poured on top of a Czapek agar plate, gently shaken by hand in a radial/rocking manner, and allowed to solidify. Afterwards, 5 µL overnight culture of candidate bacterial isolates was inoculated on top of the agar plate. 16 candidate isolates were able to be tested per plate via high-throughput screening. Plates were sealed with parafilm and cultured at 28 °C. Any inhibitory action by the bacterial isolates against *V. dahliae* was revealed by the appearance of a zone of inhibition on the agar plate. The size of the zone of inhibition was recorded at 72 h post inoculation and inhibitory action was qualitatively determined. For those isolates that produced a visible zone of inhibition, additional confirmational assays were performed using other highly virulent strains of *V. dahliae*, including V07DF2, V08DF2 and V15QY1.

### 16S rRNA identification and species designation

16S rDNA was amplified using the primer pair 27F and 1492R (Additional file 1: Table S2). Sequencing results were identified by using BLAST to search the NCBI 16S rRNA database. Species was designated based on the best hit for each species in BLAST and confirmed by genome-based taxonomy by Type Strain Genome Server (https://tygs.dsmz.de) (Meierkolthoff and Goker 2019).

### Genomic sequencing and *de-novo* assembly

The genomic DNA of *Pseudomonas* strains were extracted using the DNeasy UltraClean Microbial Kit (Cat. no. 12224–50, QIAGEN). Sequencing library was generated using NEB Next Ultra II DNA Library Prep Kit for Illumina (Cat. no. E7645, NEB) following manufacturer’s recommendations and index codes were added to each sample. Briefly, genomic DNA sample was fragmented by sonication to a size of 350 bp. Then DNA fragments were end-polished, A-tailed, and ligated with the full-length adapter for Illumina sequencing, followed by further PCR amplification. After PCR products were purified by AMPure XP system (Beckman Coulter, USA), DNA concentration was measured by Qubit®3.0 Flurometer (Cat. no. Q33216, Invitrogen, USA), libraries were analyzed for size distribution by NGS 3 K assay (PerkinElmer, USA) and quantified by real-time PCR (3 nM) and sequenced by Illumina PE150 (Illumina, USA). Genomes were assembled de-novo using SPAdes software (Bankevich et al. 2012).

### RAST annotation

Annotation of genomes was performed using the RAST Server (Rapid Annotation using Subsystem Technology) (https://rast.nmpdr.org/rast.cgi) (Overbeek et al. 2014).

### Phylogenetic tree

The full-length sequences of 10 *Pseudomonas* housekeeping genes (*acsA*, *aroE*, *dnaE*, *guaA*, *gyrB*, *mutL*, *ppsA*, *pyrC*, *recA*, and *rpoB*) (Loper et al. 2012) were extracted using RAST (Rapid Annotation using Subsystem Technology). Protein alignment and phylogenetic tree generation were accomplished using MEGA software (version 6.06) (Tamura et al. 2007).

### Core genome and unique gene analysis

Core genome and unique gene analysis was completed via BLASTP using an amino acid identity cut-off of 70% and an *e*-value of 1e−5.

### Analysis of metabolites

A search for potential biocontrol genes/gene clusters was performed based on rough prediction of antibiotics and secondary metabolite gene clusters using antiSMASH (Medema et al. 2011) or BLAST with documented metabolite gene clusters as a reference.

### Gene mutation

Construction of an in-frame deletion mutant of *orf12* () from the 7-HT gene cluster in *Pseudomonas donghuensis* 22G5 was performed as has been described previously. 500-bp upstream and downstream sequences of *orf12* were amplified separately. The upstream and downstream fragments were then concatenated by overlap extension PCR and cloned into *pEX18Gm* plasmids (Jiang et al. 2016) to generate a gene replacement vector for *orf12* (*pEX18Gm-orf12*), which was then introduced into *E. coli* S17-1 λpir competent cells (Jiang et al. 2016). *E. coli* S17-1 λpir single clone carrying the *pEX18Gm-orf12* plasmid was co-cultured with *P. donghuensis* 22G5 at 28 °C for 24 h for conjugation. Single *P. donghuensis* 22G5 transformants were selected on LB agar plates using 25 μg/mL chloramphenicol (*P. donghuensis* 22G5 could grow on the chloramphenicol-containing LB agar plate but *E. coli* S17-1 λpir could not) and 50 μg/mL gentamicin. Single transformants were then incubated without antibiotic overnight at 28 °C in 200 μL of liquid LB medium in a 96-well plate to complete the second step of allelic exchange. Serially diluted cultures were incubated at 28 °C on LB agar plates with 5% sucrose. Validity of the final *orf12* mutants from 22G5 was verified via PCR (Wang et al. 2015) and sequencing. All primers used for vector construction and PCR verification are listed in Additional file 1: Table S2.

### 7-hydroxytropolone purification

7-hydroxytropolone (7-HT) purification was performed as described (Jiang et al. 2016). *P. donghuensis* 22G5 single clone was cultured in 3 mL MKB media overnight, which was then inoculated at a 1:100 ratio to 50 mL MKB media and cultured for 48 h. 1/100 volume of hydrochloric acid (37.5%) was added to the supernatant of *P. donghuensis* 22G5 culture to adjust the pH to 2, which was then extracted using 25 mL ethyl acetate for twice, NaCl (1:10 w/v, ~ 5 g) was added to reduce the formation of ethyl acetate-water emulsion. The organic phase which containing 7-HT was rotary-evaporated to dryness under vacuum. The residue was dissolved in 10 mL ethanol and purified using a Sephadex LH20 column (Cat. no. 17-0090-02, GE) eluted by ethanol. Fractions positive in CAS assay (Schwyn and Neilands 1987) were collected and dried by rotary evaporation, and were dissolved in 1 mL DMSO and stored at − 20 °C.

### Planta in-vivo assay

*V. dahliae* strains were cultured in Czapek liquid at 28 °C for 7 days to OD_600_ = 2 (~ 3 × 10^6^ CFU/mL). *Pseudomonas protegens* XY2F4 and *P. donghuensis* 22G5 were cultured in LB liquid at 28 °C for 24 h to OD_600_ = 2 (~ 1 × 10^8^ CFU/mL). One seedling for each pot (5 cm × 5 cm square pot) was planted. One-week-old cotton seedlings in the experimental (XY2F4- or 22G5-protected) group was treated with a soil drench using a mixture of 50 mL V*. dahliae* conidia (OD_600_ = 0.2, equals approximately 3 × 10^5^ colony forming unit, CFU/mL) and 50 mL 1:10 diluted XY2F4 or 22G5 overnight culture (OD_600_ = 0.2, equals approximately 1 × 10^7^ CFU/mL). The control group was treated with a mixture of 50 mL V*. dahliae* conidia (OD_600_ = 0.2, equals approximately 3 × 10^5^ CFU/mL) and 50 mL LB culture media. Plants were monitored after inoculation for disease phenotype over time and DI (Disease Index) was calculated 30 days post-infection. The disease grade was classified as 0 (no wilting), 1 (0–25% defoliated leaves), 2 (25–50% defoliated leaves), 3 (50–75% defoliated leaves), or 4 (75–100% defoliated leaves) (Zhang et al. 2017). DI (Disease Index) is calculated as follows:

$$ DI = \frac{{\sum {\left( {{\text{d}}c \times nc} \right)} }}{nt \times 4} \times 100 $$

dc: disease severity rating; nc: number of plants in each category of disease severity; nt: total number of plants assessed (Zhao et al. 2014). 15–20 cotton seedlings were used for each treatment. At least four independent biological replicates for each treatment were performed.

### Statistical analyses

All statistical analysis was performed with the software Graphpad Prism (GraphPad Software, La Jolla, CA, USA). The Student’s *t*-test, Mann–Whitney U-test was used whenever appropriate. The *p-*values of less than 0.05 were considered statistically significant.

### Public acession of culture

*P. protegens* XY2F4 and *P. donghuensis* 22G5 were deposited in China General Microbiological Culture Collection Center (CGMCC), the CGMCC No.18017 for *P. protegens* XY2F4 and CGMCC No.18084 for *P. donghuensis* 22G5.

### NCBI accession

*P. protegens* XY2F4 (PIZE00000000), *P. donghuensis* 22G5 (RWIB00000000), *Pseudomonas putida* 25E1 (WSSD00000000), *Pseudomonas lini* 25D11 (RSFR00000000).

## Results

### High-throughput screening for isolates with inhibitory action against *V. dahliae*

**Fig. 1:**
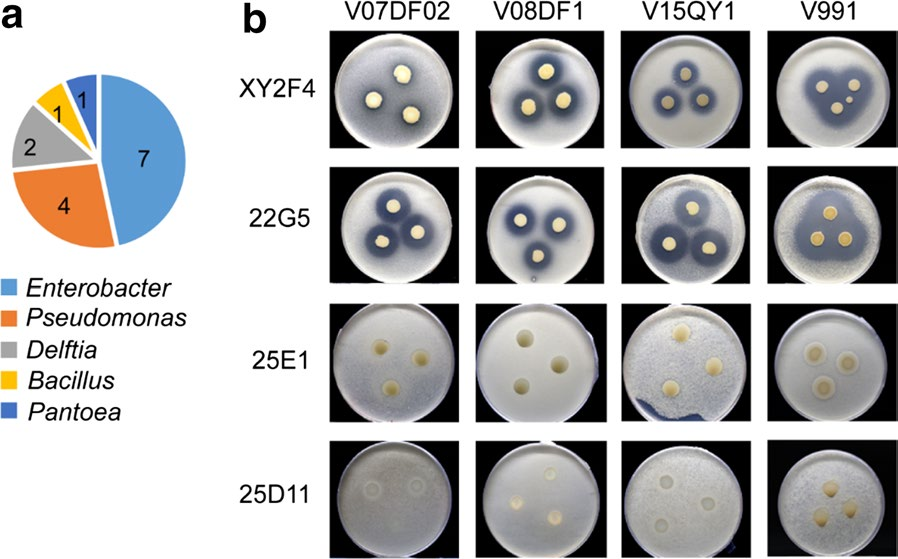
High-throughput screening of isolates with inhibitory action against *V. dahliae*. **a** Species distribution of isolates with inhibitory action against *V. dahliae*. Species were designated according to similarity to 16S rDNA. **b** Zone of inhibition assay of strains XY2F4 and 22G5 co-inoculated with conidia of various *V. dahliae* strains

Bacterial isolates recovered from 886 plant rhizosphere samples taken from the three main cotton-producing areas of China (the Yangtze river basin, the Yellow River basin and Xinjiang) (Additional file 1: Table S1) were cultured in order to determine their effect against *V. dahliae*. On average, 10 individually isolated bacterial colonies (i.e. single colonies with different color, morphologies) were tested per sample. In total, 8,736 bacterial isolates were tested in a high-throughput zone of inhibition assay (Additional file 1: Fig. S1). Isolates were considered to have shown inhibitory action against *V. dahliae* if a clear inhibition zone was observed upon co-incubation with conidia of *V. dahliae*. Of the total samples tested, 15 isolates from various bacterial genera were identified as conferring inhibitory effect on *V. dahliae* (Fig. 1a). Two strains (XY2F4 and 22G5) with significant, broad spectrum inhibitory action against multiple highly virulent *V. dahliae* strains (Fig. 1b) were selected for further genotyping. Another two strains (25E1 and 25D11) with low inhibitory capacity were randomly selected to serve as negative controls (Fig. 1b). According to identification via 16S ribosomal DNA similarity and genome-based taxonomy by Type Strain Genome Server (https://tygs.dsmz.de) (Meierkolthoff and Goker 2019), *Pseudomonas protegens* XY2F4, *P. donghuensis* 22G5, *P. putida* 25E1 and *P. lini* 25D11, were designated for study.

### Comparative genomic analysis of *Pseudomonas* spp. exhibiting inhibitory action against *V. dahliae*

**Fig. 2:**
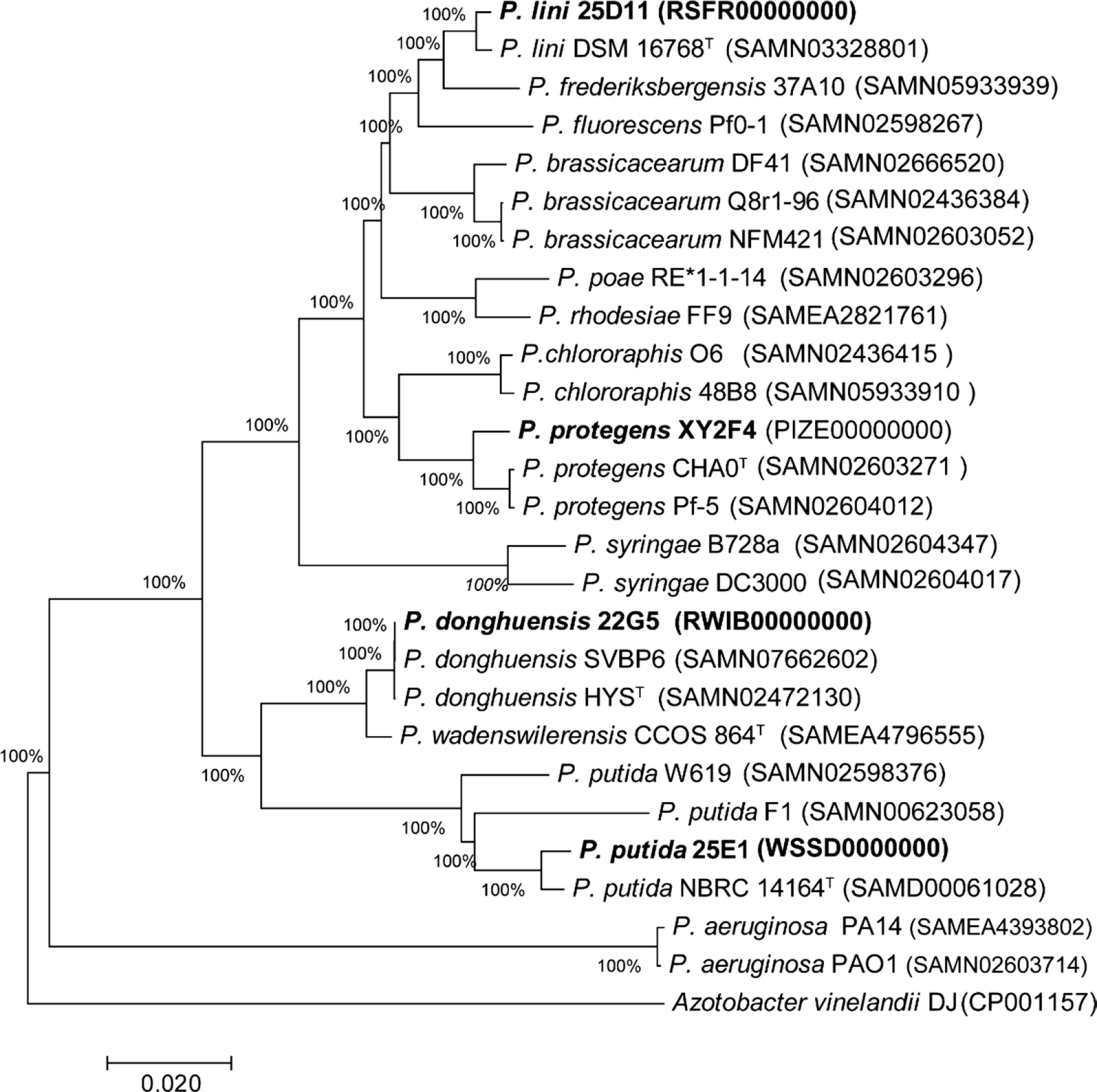
Phylogenetic tree depicting the evolutionary relationships of *Pseudomonas* spp. Genomes of previously reported strains were downloaded from The *Pseudomonas* Genome Database (https://www.pseudomonas.com/). The annotation was performed using RAST (Overbeek et al. 2014). The phylogenetic tree was generated with MEGA software using concatenated sequences of 10 housekeeping genes (*acsA*, *aroE*, *dnaE*, *guaA*, *gyrB*, *mutL*, *ppsA*, *pyrC*, *recA*, and *rpoB*) (Loper et al. 2012). Strains in this study are indicated with a star (*). Type strains or representative strains of multiple documented *Pseudomonas* species were compiled to build the tree. *Azotobacter vinelandii* DJ was used as the outgroup

**Fig. 3:**
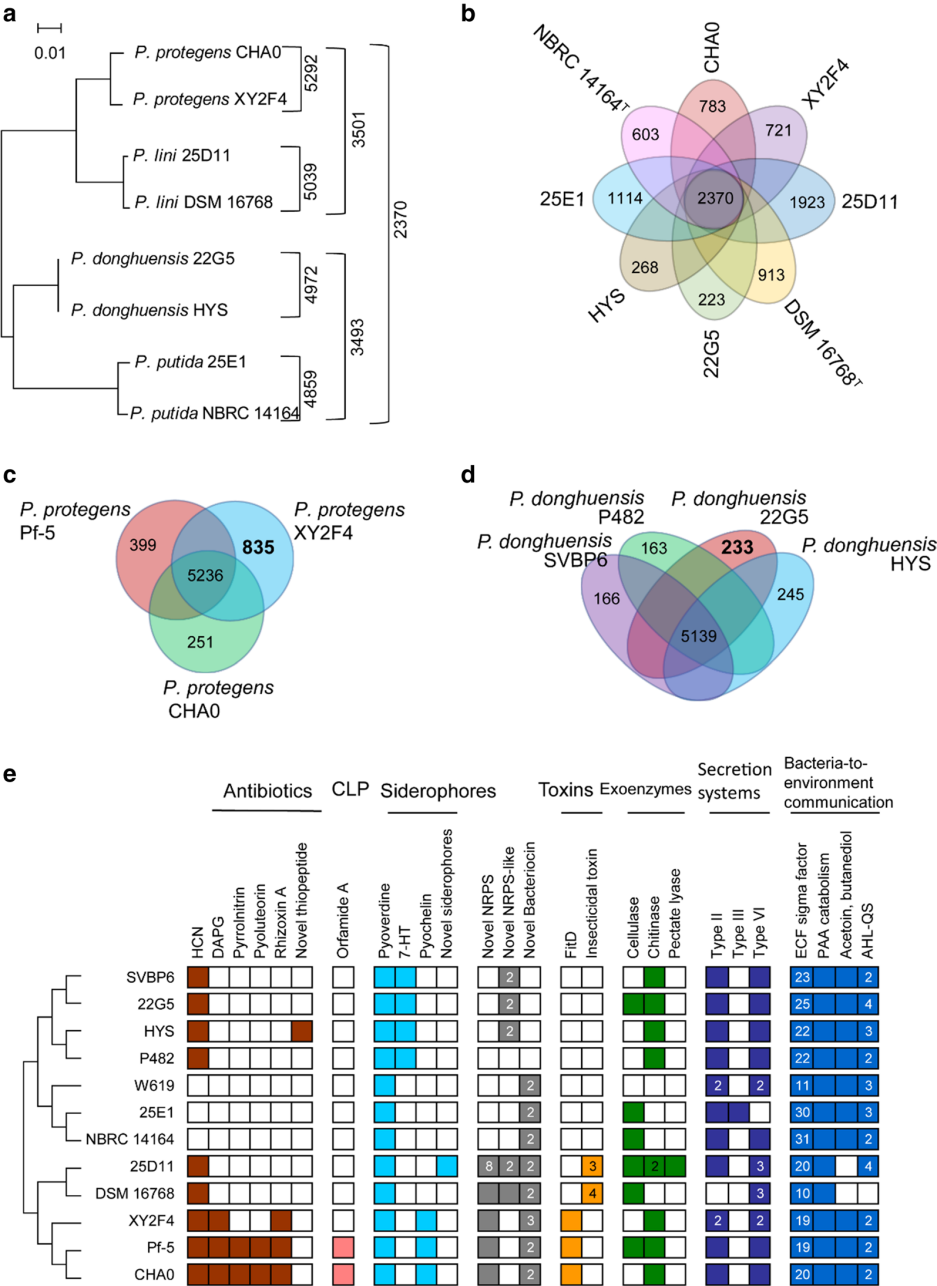
Comparative genome analysis of *Pseudomonas* strains. **a**, **b** Number of shared genes and unique genes in genomes and of *P. protegens* XY2F4, *P. donghuensis* 22G5, *P. putida* 25E1 and *P. lini* 25D11 and their type strains. **c**,** d** Number of unique genes in *P. protegens* XY2F4XY2F4 and *P. donghuensis* 22G5 when compared with published strains from the same species (with NCBI accessions). **e** Presence of selected gene clusters for biosynthesis of antibiotics, cyclic lipopeptides (CLP), siderophores, insecticidal toxins, exoenzymes, secretion systems, and chemicals for bacteria-to-environment communication. Colored box/blank box indicates the presence/absence of selected gene clusters, respectively. Numbers within boxes represent the predicted number of gene clusters encoded for the corresponding products

Whole genome sequencing and de novo assembly of the four *Pseudomonas* strains was performed (Additional file 1: Table S3). A phylogenetic tree depicting the evolutionary relationships among these four *Pseudomonas* strains and other well documented strains indicated that *Pseudomonas* isolates from different ecological environments were highly diverse (Fig. 2). From the results, *P. protegens* XY2F4 and *P. lini* 25D11 are clustered in the large group of *Pseudomonas fluorescens* “complex” (Garridosanz et al. 2016) according to the taxonomy analysis (Fig. 2), which have been taxonomically assigned to more than fifty different species, many of which have been described as plant growth-promoting rhizobacteria (PGPR) (Garridosanz et al. 2016). *P. donghuensis* 22G5 and *P. putida* 25E1 are close-related and clustered in the *P. putida* group (Fig. 2)*.* Furthermore, we compared the genes specific to each strain and genes shared among strains. Four type strains, including *P. protegens* CHA0 (Shaukat and Siddiqui 2003), *P. donghuensis* HYS (Chen et al. 2018), *P. putida* NBRC 14164^ T^ (Ohji et al. 2014), and *P. lini* DSM 16768^ T^ (Kaminski et al. 2018), as reference strain for *P. protegens* XY2F4, *P. donghuensis* 22G5, *P. putida* 25E1 and *P. lini* 25D11, respectively, are incorporated together in the analysis. These eight genomes shared a core genome of 2370 genes (Fig. 3a), and with the number of genes unique to each strain ranged from 223 to 1923 (Fig. 3b). We further compared *P. protegens* XY2F4 and *P. donghuensis* 22G5 genomes respectively with more published strains in the same species, including two well-documented *P. protegens* strains (Paulsen et al. 2005; Shaukat and Siddiqui 2003) and three published *P. donghuensis* strains to date (Chen et al. 2018; Muzio et al. 2020; Ossowicki et al. 2017). Results indicated that the *P. protegens* XY2F4 genome had 835 specific genes compared with *P. protegens* CHA0 and Pf-5 (Fig. 3c), mainly including genes for widespread colonization island, general secretion pathway, orphan regulatory proteins, and so on (Additional file 1: Table S4). The *P. donghuensis* 22G5 genome had 233 unique genes compared with three published *P. donghuensis* strains (Fig. 3d), mainly including genes in iron siderophore sensor & receptor system, n-phenylalkanoic acid degradation, fatty acid metabolism cluster, and so on (Additional file 1: Table S5).

To identify the genomic features accounting for inhibitory action against *V. dahliae*, gene clusters related to biocontrol traits and environmental interactions were compared. The biosynthesis of antibiotics, cyclic lipopeptides (CLP), siderophores, insecticidal toxins, exoenzymes, secretion systems, and chemicals for environmental communication and acyl-homoserine lactone (AHLs) mediated quorum-sensing was discovered. For comparison, multiple well-documented strains in the same species taxon were included in the analysis. Results indicated that genes related to the siderophore pyoverdine, type II secretion system (T2SS), type VI secretion system (T6SS), extracytoplasmic function (ECF) sigma factors for detecting environmental cues (Kwak et al. 2018), phenylacetic acid (PAA) catabolism, acetoin, butanediol catabolism for bacteria-plant communication (Mhlongo et al. 2018), and AHLs-mediated quorum-sensing were commonly distributed in the strains we analyzed (Fig. 3e). In addition to these, specific functional gene clusters were identified in the *V. dahliae-*inhibitory strains. 2,4-DAPG and a nonribosomal peptide synthetase (NRPS)-type gene cluster responsible for rhizoxin A, pyochelin, and FitD insecticidal toxin were specifically distributed in the *P. protegens* strains XY2F4 and Pf-5. However, XY2F4 had lost the gene clusters for biosynthesis of pyrrolnitrin, pyoluteorin and orfamide A, in compare with CHA0 and Pf-5 (Fig. 3e). A siderophore-type gene cluster involved in the biosynthesis of 7-hydroxytropolone (7-HT) was specifically identified in the *P. donghuensis* species. Gene organization in this 7-HT biosynthesis gene cluster was the same as has been previously reported in *P. donghuensis* HYS (Chen et al. 2018), a first reported *Pseudomonas* strain containing a 7-HT gene cluster. In addition, *P. donghuensis* 22G5 produces cellulase (Fig. 3e), which was found to be absent in *P. donghuensis* HYS and may function by degrading the cell walls of pathogens or by triggering plant defenses. In summary, the *Pseudomonas* strains screened out via *V. dahliae* inhibition assay have developed specific genomic characteristics that could produce certain secondary metabolites that confer inhibitory to *V. dahliae*.

### 7-HT is a major factor in *P. donghuensis* 22G5 accounting for inhibitory action against *V. dahliae*

**Fig. 4.**
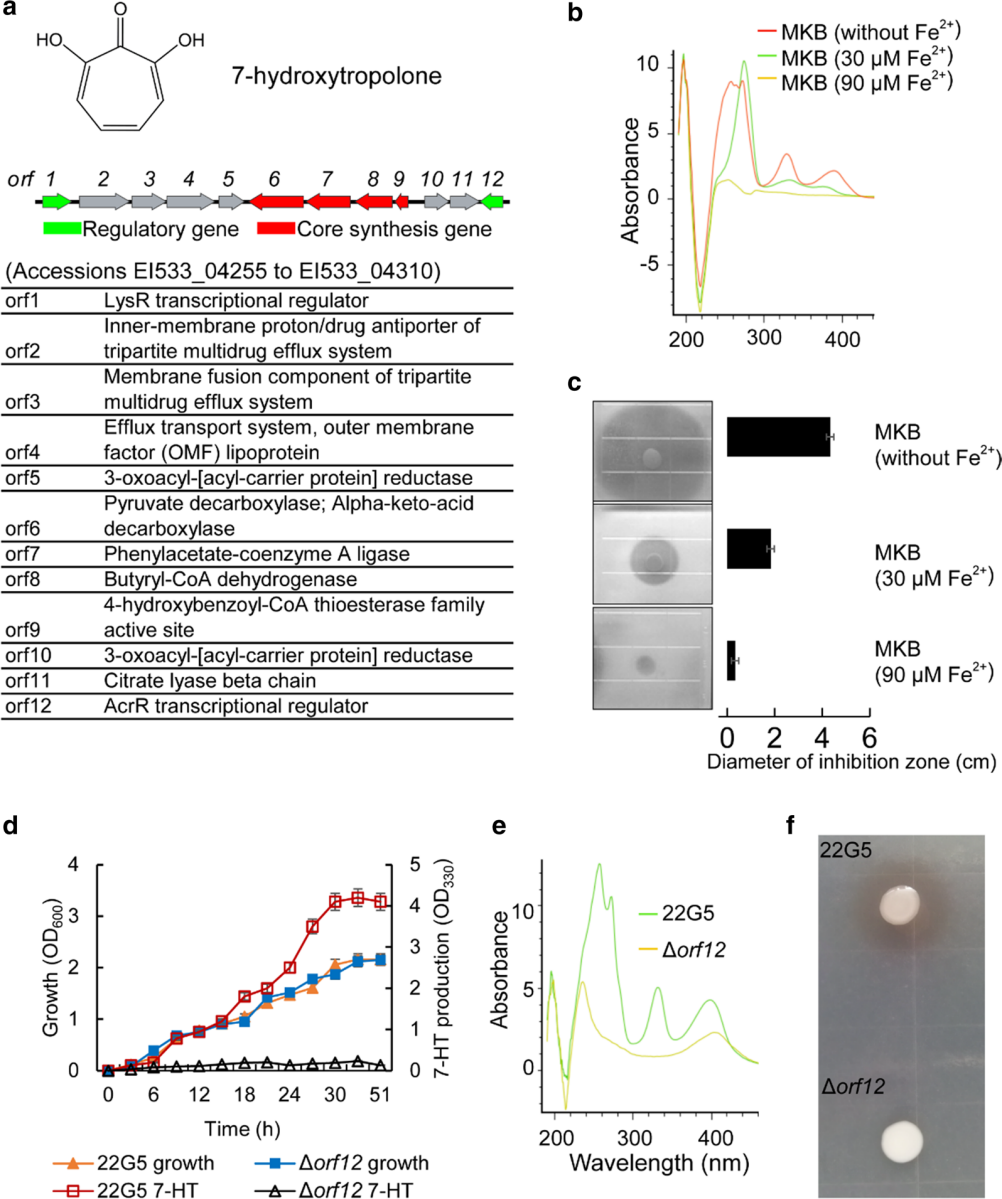
7-HT biosynthesis and inhibition of *V. dahliae*. **a** Gene cluster for 7-HT (Jiang et al. 2016). **b**, **c** Absorption spectra of 7-HT production and phenotypes in zone of inhibition assay using 7-HT extraction from the supernatant of 22G5 culture in MKB media with or without FeSO_4_. **d** Growth trace of wild type 22G5 (22G5) and *orf12* mutant (Δ*orf12*) (displayed by OD_600_, left y-axis) and its corresponding 7-HT production (displayed by OD_330_, right y-axis). **e**, **f** Absorption spectra of 7-HT production and phenotypes in zone of inhibition assay in wild type 22G5 (22G5) and *orf12* mutant (Δ*orf12*)

*P. protegens* XY2F4 has a gene cluster for biosynthesis of a biocontrol ingredient 2,4-DAPG (Fig. 3e), which is well documented in its type strain *P. protegens* CHA0 *and* Pf-5*. P. donghuensis* 22G5 has a gene cluster for biosynthesis of specific siderophore metabolite 7-HT, a metabolite common to *P. donghuensis* species which was previously reported to have fungal antagonism activity (Chen et al. 2018; Muzio et al. 2020; Ossowicki et al. 2017). We inferred that 7-HT with broad spectrum might account for their inhibitory action against *V. dahliae*. The same 7-HT biosynthesis cluster as described is composed of 12 ORFs (NCBI accessions EI533_04255 through EI533_04310). *ORF6* through *ORF9* (NCBI accessions EI533_04270 through EI533_04285) encode the core biosynthesis genes while *ORF1* (NCBI accession EI533_04310) and *ORF12* (NCBI accession EI533_04255) are regulatory genes (Fig. 4a). Supernatant derived from 22G5 cultures showed characteristic absorption peaks at 330 nm and 392 nm, which was identical to 7-HT (Jiang et al. 2016). As a siderophore, 7-HT’s biosynthesis was regulated by iron concentration in the culture media. Consistently, we found that the yield of 7-HT in 22G5 cultures declined dramatically as iron (FeSO_4_) concentration increased in the MKB media (Fig. 4b), suggesting the existence of a regulatory feedback mechanism for biosynthesis of 7-HT based on growth conditions of high iron concentration. The 7-HT was then extracted and purified from supernatant retrieved from 22G5 cultures and applied to a plate assay using *V. dahliae*. As expected, the inhibition zone shrunk upon reduction of 7-HT in the MKB medium (Fig. 4c). Next we generated a gene mutation of *ORF12* via homologous recombination (Additional file 1: Fig. S2). As a result, the *ORF12-*mutated strain (Δ*orf12*) did not show much differences in growth rate compared with wild type 22G5 (Fig. 4d), however, lost its ability to produce 7-HT (Fig. 4e), and phenotypically, lost the ability to inhibit *V. dahliae* (Fig. 4f). With these tests, we demonstrated that 7-HT originating from *P. donghuensis* 22G5 is the major factor accounting for inhibitory effects against *V. dahliae*.

### *In planta* assays showed *Pseudomonas* strains XY2F4 and 22G5 significantly improve resistance to cotton verticillium wilt

**Fig. 5:**
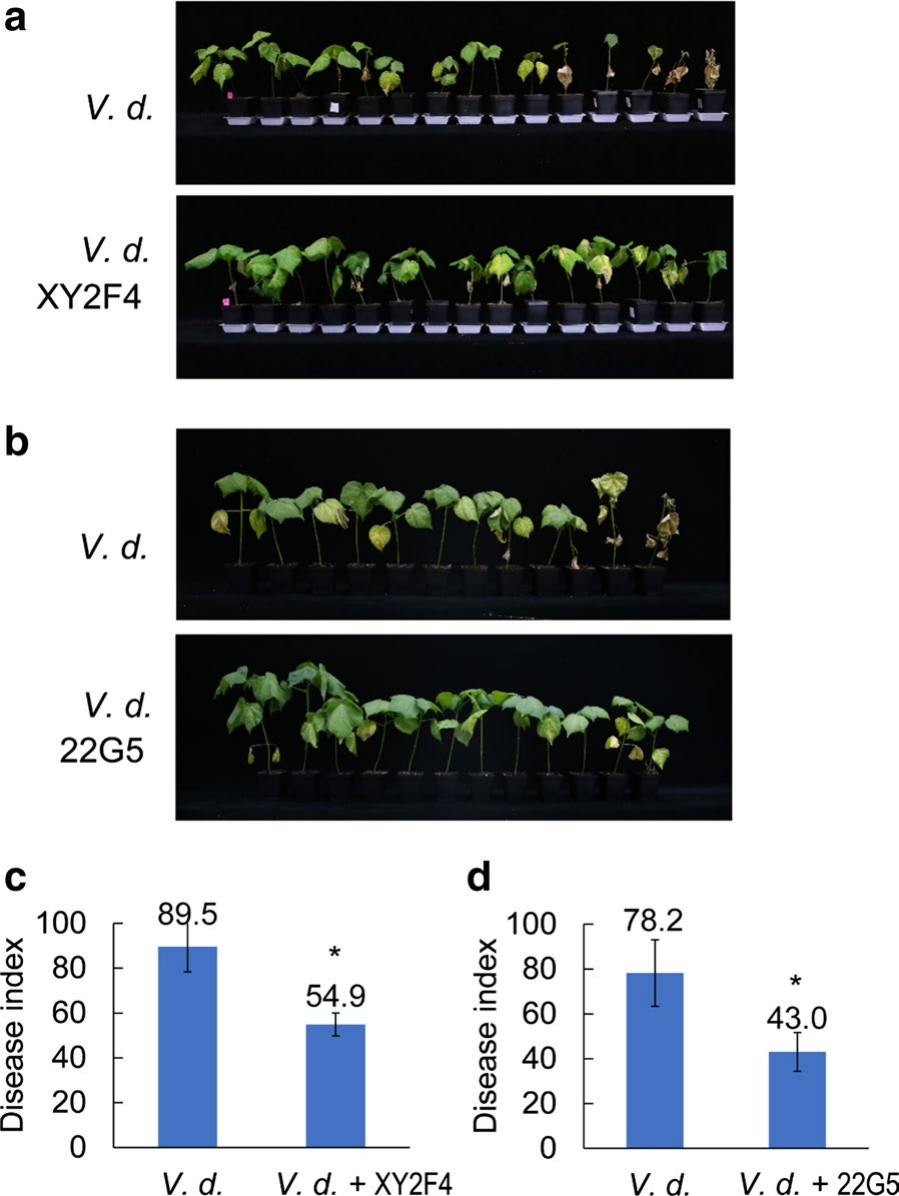
*Pseudomonas* strains XY2F4 and 22G5 significantly improve cotton verticillium wilt resistance. **a**, **b** Phenotypes of *G. hirsutum* acc. Texas Marker-1 (TM-1) treated with a mixture of *V. dahliae* conidia (*V. d.*) and culture of XY2F4 (**a**) or 22G5 (**b**), and control group treated with *V. dahliae* conidia only. Plants were monitored after inoculation for disease phenotype over time, and DI (Disease Index) was calculated as has been described previously (Zhao et al. 2014) 30 days post-infection. See materials and methods for details. **c**, **d** DI (Disease Index) summarized from four independent replicates of planta in-vivo assays as described in panel **a**, **b**. Mann–Whitney Non-parametric Test. *P* < 0.05

To evaluate whether the strains screened via plate assay also showed potential for biocontrol in host–pathogen interaction, planta in vivo assays were conducted using *P. protegens* XY2F4 and *P. donghuensis* 22G5. Plant growth promotion or biosafety tests for XY2F4 and 22G5 were performed first using three different concentration of culture (1 × 10^7^, 1 × 10^6^ and 1 × 10^5^ CFU/mL) by soil drench. Results indicated that pre-treatment with *P. protegens* XY2F4 cultures (1 × 10^7^ CFU/mL) increased seedling biomass (Additional file 1: Fig. S3, *P* < 0.05). In contrast, *P. donghuensis* 22G5 cultures had no significant effect on plant biomass compared with the LB media control group (CK), indicating that it poses low biosafety risk (i.e. non-pathogenic) to cotton plants. Planta in vivo assays were also conducted using cotton seedlings co-inoculated with *V. dahliae* and the *Pseudomonas* strains with *V. dahlia* inhibitory activity in plate assay. Combinations of *V. dahliae* conidia and either *P. protegens* XY2F4 or *P. donghuensis* 22G5 were tested. It was observed 30 days after inoculation that cotton seedlings co-inoculated with either XY2F4 or 22G5 strains and *V. dahliae* conidia exhibited a less degree of leaf chlorosis, necrosis and wilting than seedlings inoculated with *V. dahliae* V991 alone (Fig. 5a, b). The disease index of XY2F4- and 22G5-protected cotton seedlings of upland cotton cultivar TM-1 was significant reduced that summarized from 4 independent biological replicates, compared to the un-protected group (Fig. 5c, d). Thus, we confirmed that the *Pseudomonas* species isolated via high-throughput screening significantly protect cotton plants against VW infection.

## Discussion

### *Pseudomonas* spp. are important operational taxonomic units in the rhizosphere with antagonistic action toward *V. dahliae*

Rhizosphere microbiomes are major determinants of plant health and productivity, and they have the potential to improve sustainable agricultural practices by influencing growth, yield, nutrient uptake, and biotic/abiotic tolerance (Veach et al. 2019). Plant growth–promoting rhizobacteria (PGPR) are naturally occurring soil bacteria that aggressively colonize plant rhizospheres and benefit plants by providing growth promotion or protection against pests and pathogens (Goswami et al. 2016). Both soil conditions and plant host species are commonly recognized as important determinants of the soil microbial composition (Berg et al. 2005; Rodriguez et al. 2019). *Arabidopsis* genotypes with a manipulated systemic expression of SA signaling have been shown to have an increased population density of *Pseudomonas* spp. (Doornbos et al. 2011). The microbiome structures of tomato varieties either resistant or susceptible to the soil-borne pathogen *Ralstonia solanacearum* differ (Kwak et al. 2018), indicating that some biochemical or molecular attributes of specific plants may constitute a host-induced filter for the microbiome in the plant–soil environment. Field research has also revealed that monoculture, but not crop rotation, leads to an enrichment of bacteria producing 2,4-DAPG, a well-known antibiotic originating from *P. protegens* that suppresses soil-borne pathogens (Weller et al. 2007). Until now, multiple strains from fluorescent *Pseudomonas* spp. and *Bacillus* spp*.* were reported to have biocontrol potential against *V. dahliae* (Erdogan and Benlioglu 2010; Lan et al. 2017; Li et al. 2012b; Mercadoblanco et al. 2004; Sherzad and Canming 2020; Zhang et al. 2018). However, without a parallel comparison of various hosts, rotation methods, soil types, and races of *V. dahliae,* the effectiveness of biocontrol among these strains has been difficult to determine. Our data provide profiles of antagonistic OTUs (operational taxonomic units) from 8736 isolates recovered from the three main cotton-producing areas of China, and we have shown via *in planta* assays that certain *Pseudomonas* spp. are important antagonistic OTUs that effectively suppress *V. dahliae.*

### Genetic and metabolic diversity in *Pseudomonas* spp. reveals distinct mode of action for biocontrol of *Verticillium* wilt

The genus *Pseudomonas* belonging to the gamma subclass of proteobacteria is a group of bacteria with remarkable metabolic, genetic, and ecological diversity. It currently contains more than 100 designated species that are present in all major natural environments associated with plants and animals (Peix et al. 2018). For example, *P. aeruginosa* is an opportunistic pathogen in humans. In plants, *Pseudomonas* spp. provide promising models for plant–microbe interactions owing to the species’ metabolic, ecological, and genetic diversity (Sitaraman 2015). In the past 30 years, reference strains for different species of plant-associated *Pseudomonas* have been extensively documented for their many beneficial metabolites and ecological functions (David et al. 2018). However, a deep understanding of the modes of action underlying their biocontrol effects against *V. dahliae* is still lacking. Similar to the type strain of *P. protegens* (CHA0 and Pf-5 as type strains), *P. protegens* XY2F4 characteristically and conservatively has gene clusters for the biosynthesis of the natural phenolic antimicrobial compounds 2,4-DAPG (Nowak-Thompson et al. 1994) and rhizoxin A (Loper et al. 2008). 2,4-DAPG was commonly active against various soil-borne bacterial and fungal pathogens that cause plant diseases (Troppens et al. 2013; Yang and Cao 2012) and also showed toxicity to plant-parasitic and bacterial-feeding nematodes (Meyer et al. 2009). Moreover, 2,4-DAPG was found to mediate induced systemic resistance (ISR) by triggering the JA/ET-mediated defense system in *Arabidopsis* (Chae et al. 2020). *P. donghuensis* HYS and *P. donghuensis* 22G5 conservatively produce a novel siderophore, 7-HT. 7-HT was first reported as a siderophore in *P. donghuensis* HYS in 2016 (Jiang et al. 2016), and the gene cluster responsible for its production has been well established (Chen et al. 2018; Krzyżanowska et al. 2016). Recently, 7-HT was found to be the main metabolite responsible for the fungal antagonism of *P. donghuensis* SVBP6 by testing its antagonism activities against *Macrophomina phaseolina*, *Fusarium graminearum*, *Fusarium semitectum* and *Bacillus subtilis* (Muzio et al. 2020). The antagonism results were obtained from the growth inhibition of phytopathogenic fungi when they were co-cultured/co-inoculated with *P. donghuensis* SVBP6 or its supernatant. Iron is a necessary element in virtually all living organisms and is utilized to catalyze a wide variety of indispensable enzymatic reactions (Soares and Weiss 2015). As a siderophore, 7-HT was expected to function as an iron scavenger when interacting with phytopathogenic fungi. Our study enriched our knowledge by showing that 7-HT had broad-spectrum activity against phytopathogenic fungi, including *V. dahliae*. It was reported that 7-HT is virulent toward *C. elegans* (Gui et al. 2020); however, a possible mode of action has not been studied. In conclusion, the genetic and metabolic diversity of *Pseudomonas* spp. provides distinct modes of action that are dependent on plant–microbe interactions, allowing the biocontrol of cotton VW.

### Discovery of antagonistic *Pseudomonas* strains in rhizosphere provides promising material for development of biocontrol agents

Active management of the microbiome of agriculturally important plants promises to optimize plant reliability, the use of resources, and the environmental impact related to food production by enhancing plant growth, nutrient use efficiency, abiotic stress tolerance, and disease resistance (Busby et al. 2017). Bacterial isolates displaying inhibitory action in in vitro plate assays may not demonstrate biocontrol action in the greenhouse in *in planta* assays or field trials, because some strains may not be able to colonize the rhizosphere (the plant root surface or intercellular spaces of plants) in order to deliver their effectors (Deketelaere et al. 2017). Rotation methods, soil types, host species, and the variety of *V. dahliae* all also affect rhizosphere fitness and the efficiency of biocontrol agents (BCAs). To date, multiple strains distributed across four different species of *Pseudomonas* have been successfully developed and registered as biopesticides that are commercially available to growers for the biocontrol of many plant diseases caused by plant pathogenic fungi and bacteria, including *P. chlororaphis* 63–28, *P. aureofaciens* Tx-1, *P. fluorescens* A506, and *P. syringae* ESC-10 (Fravel 2005). *Pseudomonas* has additional promising BCAs to offer for agricultural disease management. Our data identified two more effective *Pseudomonas* strains with broad biocontrol action against various strains of *V. dahliae.* In addition, our *P. donghuensis* 22G5 showed a higher amount of 7-HT production (the OD_330_ of 7-HT produced by 22G5 reached 4 to 5 when the OD_600_ of the culture reached 2) (Fig. 4d) compared with that reported for *P. donghuensis* SVBP6 (Muzio et al. 2020) (the OD_330_ of 7-HT produced by SVBP6 reached 1 when the OD_600_ of the culture reached 2). Moreover, our data from the *in planta* assay first showed the effectiveness of *P. donghuensis* against *V. dahliae*, and this further supported its applications in the development of BCAs for VW manipulation in cotton farming. However, several perspectives are remained to be addressed: (1) the suggested application frequency and method (e.g., seed coat or soil drench) of *Pseudomonas* strains used as BCAs (Angelopoulou et al. 2014); (2) the concerted effects of multiple *Pseudomonas* strains applied as a mixture; (3) the ecological influence of BCA *Pseudomonas* strains on the distribution of other OTUs in the rhizosphere (Angelopoulou et al. 2014).

## Supplementary information

**Additional file 1: Figure S1.** Schematic diagram showing work flow for high-throughput screening. **Figure S2. **Schematic diagram illustrating the use of the pEX18Gm suicide plasmids to generate a gene deletion in *P. donghuensis *22G5. Picture was modified from the reference (Wang et al. 2015). Refer to Table S2 for detailed primer sequences. **Figure S3. **Plant growth promoting and biosafety tests of *P. protegens *XY2F4 and *P. donghuensis *22G5. 7-day old cotton seedlings with treatments of different concentrations of *Pseudomonas *strains (XY2F4 and 22G5) and the LB control (CK), biomass were recorded three weeks after inoculation. 1:10, 1:10 dilution of overnight culture, ~1 × 10^7^ CFU/mL; 1:100, ~1 × 10^6^ CFU/mL; 1:1000, ~1 × 10^5^ CFU/mL. * *P* < 0.05. Student’s t test. **Table S1. **Informations of plant rhizosphere samples. **Table S2. **Primer used in this study. **Table S3. **Genome summary of *Pseudomonas *strains. **Table S4.** RAST Annotation of unique genes in XY2F4. **Table S5.** RAST Annotation of unique genes in 22G5.

## Data Availability

All data generated or analysed during this study are included in this published article.
