# Supplementary material for: Pseudomonas species isolated via high-throughput screening significantly protect cotton plants against verticillium wilt
Source: AMB Express. 2020 Oct 28;10:193. doi: 10.1186/s13568-020-01132-1 (PMC7593376; doi:10.1186/s13568-020-01132-1)
Supplement: Supplementary file 1 — Additional file 1: Figure S1. Schematic diagram showing work flow for high-throughput screening. Figure S2. Schematic diagram illustrating the use of the pEX18Gm suicide plasmids to generate a gene deletion in P. donghuensis 22G5. Picture was modified from the reference (Wang et al. 2015). Refer to Table S2 for detailed primer sequences. Figure S3. Plant growth promoting and biosafety tests of P. protegens XY2F4 and P. donghuensis 22G5. 7-day old cotton seedlings with treatments of different concentrations of Pseudomonas strains (XY2F4 and 22G5) and the LB control (CK), biomass were recorded three weeks after inoculation. 1:10, 1:10 dilution of overnight culture, ~1 × 10^7^ CFU/mL; 1:100, ~1 × 10^6^ CFU/mL; 1:1000, ~1 × 10^5^ CFU/mL. * P < 0.05. Student’s t test. Table S1. Informations of plant rhizosphere samples. Table S2. Primer used in this study. Table S3. Genome summary of Pseudomonas strains. Table S4. RAST Annotation of unique genes in XY2F4. Table S5. RAST Annotation of unique genes in 22G5. [file 13568_2020_1132_MOESM1_ESM.pdf]

AMB Express

Pseudomonas species isolated via high-throughput screening significantly protect cotton plants against verticillium wilt

Xiaoyuan Tao[1], Hailin Zhang[1], Mengtao Gao[2], Menglin Li[2], Ting Zhao[1], Xueying Guan[1]*

[1] College of Agriculture and Biotechnology, Zhejiang University, Hangzhou, 310058, China

[2] State Key Laboratory of Crop Genetics and Germplasm Enhancement, Cotton Hybrid R & D Engineering Center (the Ministry of Education), College of Agriculture, Nanjing Agricultural University, Nanjing 210095, China.

* Corresponding author. Correspondence should be addressed to X. Guan (xueyingguan@zju.edu.cn)

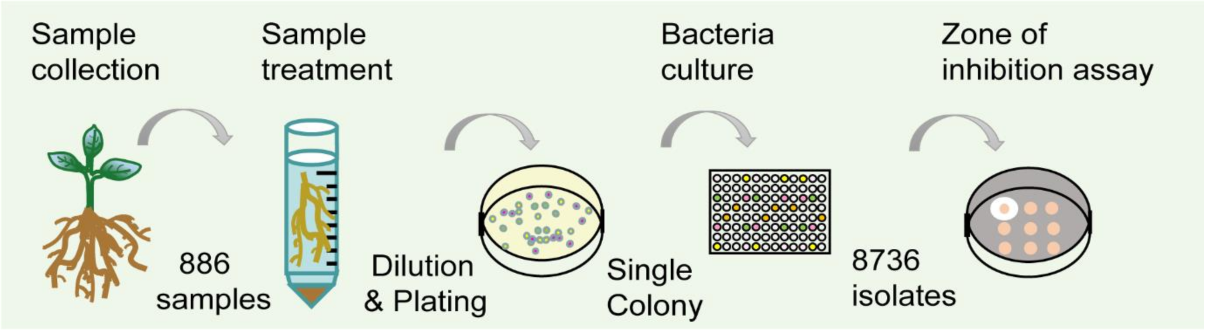

Figure S1. Schematic diagram showing work flow for high-throughput screening.

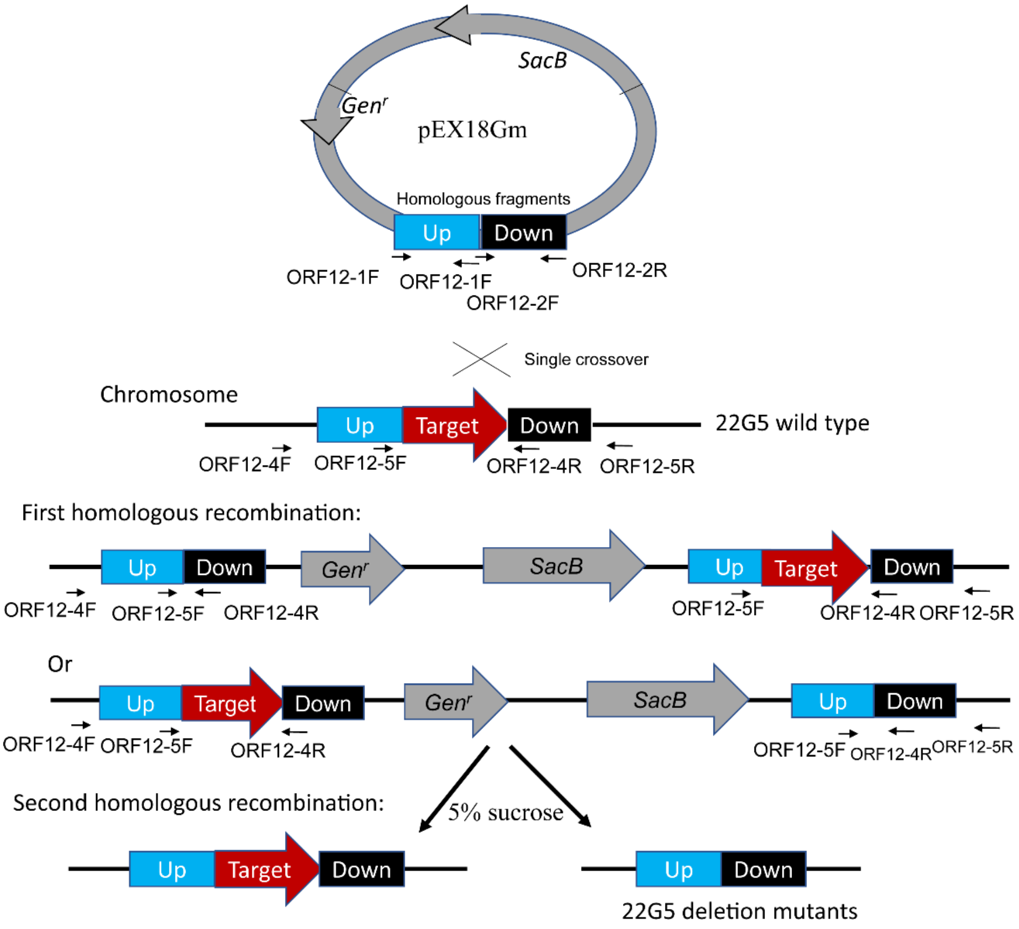

Figure S2. Schematic diagram illustrating the use of the pEX18Gm suicide plasmids to generate a gene deletion in *P. donghuensis* 22G5. Picture was modified from the reference (Wang et al. 2015). Refer to Table S2 for detailed primer sequences.

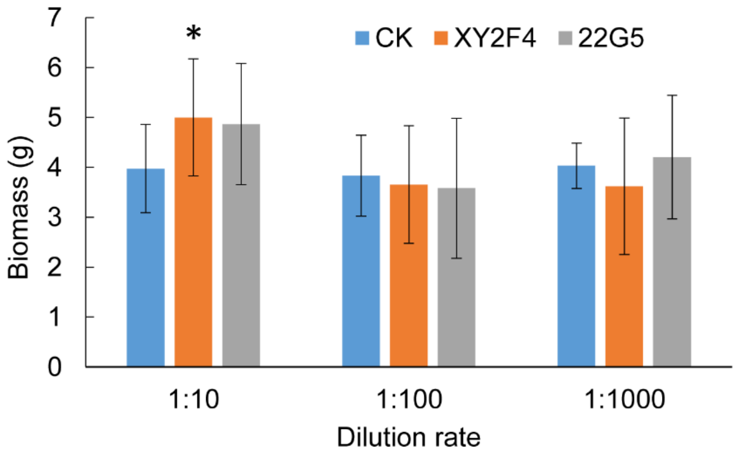

Figure S3. Plant growth promoting and biosafety tests of *P. protegens* XY2F4 and *P. donghuensis* 22G5. 7-day old cotton seedlings with treatments of different concentrations of *Pseudomonas* strains (XY2F4 and 22G5) and the LB control (CK), biomass were recorded three weeks after inoculation. 1:10, 1:10 dilution of overnight culture, ~1×10^7 CFU/mL; 1:100, ~1×10^6 CFU/mL; 1:1000, ~1×10^5 CFU/mL. * $P<0.05$. Student's t test.

Table S1 Informations of plant rhizosphere samples.

Location	Plant type	Collection time (year.month)	Plant rhizosphere samples No.	96 plate No.
Dangtu, Anhui	Cotton & Weeds & Rice	2107.6	1-60	1-7
Dangtu, Anhui	Tea	2017.6	61-77	8-9
Hangzhou, Zhejiang	Soybean	2107.6	78-150	10-14
Hangzhou, Zhejiang	Tea	2017.6	151-156	
Shihezi, Xinjiang	Cotton	2017.7	157-183	15-21
Dangtu, Anhui	Cotton & Weeds	2107.7	184-202	
Baihe, Jilin	Weeds	2017.8	203-217	22-23
Dongyan, Zhejiang	Sorghum, Sesame	2017.8	218-261	24-27
Zhangjiajie, Hebei	Weeds	2017.8	262-275	
Dangtu, Anhui	Cotton	2017.9	276-288	28-29
Zhoushan, Zhejiang	Weeds	2107.9	289-328	30-34
Haerbing, Heilongjiang	Weeds	2017.9	329-361	35-37
Suzhou, Jiangsu	Weeds	2017.10	376-373	38-43
Yancheng, Shandong	Weeds	2017.10	374-385	
Xuzhou, Jiangsu	Cotton & Weeds	2017.10	386-413	
Dangtu, Anhui	Cotton & Weeds & Rice	2017.11	414-846	44-88
Shanghai	Cotton	2018.4	847-886	89-91

Table S2. Primer used in this study.

Name	Sequence (5'-3')	Usage
27F	AGAGTTTGATCMTGGCTCAG	16S rDNA
1492R	TACGGYTACCTTGTTACGACTT	
ORF12-1F	TGTACGGGTCATGGAAGTGCA	Gene mutation,for upstream fragment
ORF12-1R	ttacctgcaatcacgTCTTTGAACCGGCAG	PCR
ORF12-2F	CTGCCGGTTCAAAGAcgtgattgcaggtaa	Gene mutation,for downstream fragment PCR
ORF12-2R	TGATCGAGCTGGACAAGCAGA	
ORF12-3F	cccggatccTGTACGGGTCATGGAAGTGCA	Gene mutation, for upstream plus downstream fragment PCR
ORF12-3R	cccaagcttTGATCGAGCTGGACAAGCAGA	
ORF12-4F	GCAGCCTCGGGTTTCACCGCCCGCCAGCTG	For first allelic exchange PCR identification
ORF12-4R	TCGAACAAGCGGCCAGTACCGGCAGTGTGC	
ORF12-5F	TCGCCAAAGGCGTGCTCGATGCCGTGCAGA	For first allelic exchange PCR identification
ORF12-5R	TGCGGGCGGATCAGCTCGGTGCGCATCAAC	
ORF12-6F	TGTACGGGTCATGGAAGTGC	For second allelic exchange PCR identification
ORF12-6R	AGATTGAACACTGGCAGGGG	
ORF12-7F	CTGTTTGCCTTACTGCGACG	For second allelic exchange PCR identification
ORF12-7R	CCTGGCGATGCTCTAGTTGT	

Table S3 Genome summary of *Pseudomonas* strains.

	P. protegens XY2F4	*P. donghuensis* 22G5	*P. lini* 25D11	*P. putida* 25E1
Genome size (bp)	6,811,381	5,726,777	7,870,958	6,975,274
Number of contings	50	51	4451	1205
GC content (%)	63.6	62.2	58.7	61.2
CDS	6153	5339	10361	7140
Average CDS size (bp)	969	964	661	823
rRNA	8	4	5	4
tRNA	60	60	71	84

Table S4 RAST Annotation of unique genes in XY2F4.

Feature	Counts
Widespread_colonization_island	10
General_Secretion_Pathway	9
Orphan_regulatory_proteins	5

Methionine_Biosynthesis	4
Rhamnose_containing_glycans	4
Biphenyl_Degradation	3
Butanol_Biosynthesis	3
Cinnamic_Acid_Degradation	3
Cresol_degradation	3
Glutathione:_Non-redox_reactions	3
Lacto-N-Biose_I_and_Galacto-N-Biose_Metabolic_Pathway	3
Lactose_and_Galactose_Uptake_and_Utilization	3
N-linked_Glycosylation_in_Bacteria	3
Polyamine_Metabolism	3
Restriction-Modification_System	3
Universal_stress_protein_family	3
5-FCL-like_protein	2
Bacterial_hemoglobins	2
Biotin_biosynthesis	2
Capsular_heptose_biosynthesis	2
Chitin_and_N-acetylglucosamine_utilization	2
Conserved_gene_cluster_possibly_involved_in_RNA_metabolism	2
D-galactarate,_D-glucarate_and_D-glycerate_catabolism	2
Fatty_acid_metabolism_cluster	2
Fermentations:_Lactate	2
Fermentations:_Mixed_acid	2
Glutamine,_Glutamate,_Aspartate_and_Asparagine_Biosynthesis	2
Glycerate_metabolism	2
Glycerolipid_and_Glycerophospholipid_Metabolism_in_Bacteria	2
Maltose_and_Maltodextrin_Utilization	2
Methylglyoxal_Metabolism	2
NAD_and_NADP_cofactor_biosynthesis_global	2
n-Phenylalkanoic_acid_degradation	2
Pentose_phosphate_pathway	2
Phd-Doc,_YdcE-YdcD_toxin-antitoxin_(programmed_cell_death)_systems	2
Putrescine_utilization_pathways	2
Ton_and_Tol_transport_systems	2
Toxin-antitoxin_replicon_stabilization_systems	2
Two_partner_secretion_pathway_(TPS)	2
Type_I_Restriction-Modification	2
Acetyl-CoA_fermentation_to_Butyrate	1
Allantoin_Utilization	1
Amidase_clustered_with_urea_and_nitrile_hydratase_functions	1
Archaeal_lipids	1
Benzoate_transport_and_degradation_cluster	1
Broadly_distributed_proteins_not_in_subsystems	1
Butyrate_metabolism_cluster	1
carbazol_degradation_cluster	1
Carbon_monoxide_oxidation	1
Cell_division-ribosomal_stress_proteins_cluster	1
Central_meta-cleavage_pathway_of_aromatic_compound_degradation	1
Chorismate_Synthesis	1

CMP-N-acetylneuraminate_Biosynthesis	1
Coenzyme_M_biosynthesis	1
Colanic_acid_biosynthesis	1
Common_Pathway_For_Synthesis_of_Aromatic_Compounds_(DAHP_synthase_to_chorismate)	1
Copper_homeostasis	1
Creatine_and_Creatinine_Degradation	1
Cyanophycin_Metabolism	1
Cysteine_Biosynthesis	1
D-gluconate_and_ketogluconates_metabolism	1
dTDP-rhamnose_synthesis	1
Ethylmalonyl-CoA_pathway_of_C2_assimilation	1
Fosfomycin_resistance	1
Gentisate_degradation	1
Glutamate_and_Aspartate_uptake_in_Bacteria	1
Glutathione:_Biosynthesis_and_gamma-glutamyl_cycle	1
Iron_siderophore_sensor_&_receptor_system	1
Lactose_utilization	1
L-rhamnose_utilization	1
Lysine_degradation	1
Lysine_fermentation	1
Magnesium_transport	1
Mannose_Metabolism	1
Methionine_Degradation	1
O-Methyl_Phosphoramidate_Capsule_Modification_in_Campylobacter	1
Oxidative_stress	1
Phosphate-binding_DING_proteins	1
p-Hydroxybenzoate_degradation	1
Poly-gamma-glutamate_biosynthesis	1
Polyhydroxybutyrate_metabolism	1
Proline,_4-hydroxyproline_uptake_and_utilization	1
Purine_Utilization	1
Quinate_degradation	1
Respiratory_dehydrogenases_1	1
Ribonucleases_in_Bacillus	1
Salicylate_and_gentisate_catabolism	1
Siderophore_Achromobactin	1
Siderophore_Aerobactin	1
Soluble_cytochromes_and_functionally_related_electron_carriers	1
Threonine_and_Homoserine_Biosynthesis	1
Transcription_factors_bacterial	1
Transcription_initiation,_bacterial_sigma_factors	1
Trans-envelope_signaling_system_VreARI_in_Pseudomonas	1
Translation_termination_factors_bacterial	1
tRNA_aminoacylation,_Asp_and_Asn	1
tRNA_aminoacylation,_Cys	1
tRNA_aminoacylation,_Glu_and_Gln	1
tRNA_aminoacylation,_Val	1
Utilization_of_glutathione_as_a_sulphur_source	1
Xanthine_dehydrogenase_subunits	1

Table S5 RAST Annotation of unique genes in 22G5.

Feature	Counts
Type_I_Restriction-Modification	2
Restriction-Modification_System	2
n-Phenylalkanoic_acid_degradation	2
Leucine_Degradation_and_HMG-CoA_Metabolism	2
Iron_siderophore_sensor_&_receptor_system	2
Fatty_acid_metabolism_cluster	2
CMP-N-acetylneuraminate_Biosynthesis	2
Rhamnose_containing_glycans	1
pVir_Plasmid_of_Campylobacter	1
Polyhydroxybutyrate_metabolism	1
Phage_tail_proteins	1
N-linked_Glycosylation_in_Bacteria	1
Lysine_fermentation	1
Lactose_and_Galactose_Uptake_and_Utilization	1
Lacto-N-Biose_I_and_Galacto-N-Biose_Metabolic_Pathway	1
Isoleucine_degradation	1
HMG_CoA_Synthesis	1
Glutamine,_Glutamate,_Aspartate_and_Asparagine_Biosynthesis	1
Glutamate_and_Aspartate_uptake_in_Bacteria	1
Exopolysaccharide_Biosynthesis	1
DNA_repair,_bacterial_RecFOR_pathway	1
DNA_repair,_bacterial	1
Butyrate_metabolism_cluster	1
Butanol_Biosynthesis	1
Biotin_biosynthesis	1
Acetyl-CoA_fermentation_to_Butyrate	1
